# Eye-tracking correlates of response to joint attention in preschool children with autism spectrum disorder

**DOI:** 10.1186/s12888-023-04585-3

**Published:** 2023-03-29

**Authors:** Ryan Anthony de Belen, Hannah Pincham, Antoinette Hodge, Natalie Silove, Arcot Sowmya, Tomasz Bednarz, Valsamma Eapen

**Affiliations:** 1 School of Computer Science and Engineering, University of New South Wales, Sydney, New South Wales Australia; 2 School of Psychiatry, University of New South Wales, Sydney, New South Wales Australia; 3 Children’s Hospital Westmead, Sydney, New South Wales Australia; 4 School of Medicine, University of Sydney, Sydney, New South Wales Australia; 5 School of Art & Design, University of New South Wales, Sydney, New South Wales Australia; 6 Academic Unit of Child Psychiatry South West Sydney, Ingham Institute, Liverpool Hospital, Sydney, New South Wales Australia

**Keywords:** Eye-tracking technology, Response to joint attention

## Abstract

**Background:**

A number of differences in joint attention behaviour between children with autism spectrum disorder (ASD) and typically developing (TD) individuals have previously been documented.

**Method:**

We use eye-tracking technology to assess response to joint attention (RJA) behaviours in 77 children aged 31 to 73 months. We conducted a repeated-measures analysis of variance to identify differences between groups. In addition, we analysed correlations between eye-tracking and clinical measures using Spearman’s correlation.

**Results:**

The children diagnosed with ASD were less likely to follow gaze compared to TD children. Children with ASD were less accurate at gaze following when only eye gaze information was available, compared to when eye gaze with head movement was observed. Higher accuracy gaze-following profiles were associated with better early cognition and more adaptive behaviours in children with ASD. Less accurate gaze-following profiles were associated with more severe ASD symptomatology.

**Conclusion:**

There are differences in RJA behaviours between ASD and TD preschool children. Several eye-tracking measures of RJA behaviours in preschool children were found to be associated with clinical measures for ASD diagnosis. This study also highlights the construct validity of using eye-tracking measures as potential biomarkers in the assessment and diagnosis of ASD in preschool children.

## Background

Autism spectrum disorder (ASD) is a neurodevelopmental disorder characterized by challenges in reciprocal social communication behaviours and the presence of restricted and repetitive behaviours [1]. One challenge for ASD children is their ability to accurately engage in joint attention behaviours. Joint attention involves sharing a mutual reference point (e.g., a physical or mental object) with another person, making it an essential part of human social cognition [2]. Joint attention has been extensively investigated in infants, due to its important role in the development of information processing [2] and language [3, 4] (refer to [5, 6] for a review). In the first years of life, typically developing (TD) infants respond to joint attention (RJA) by coordinating their attention with that of their primary caregivers. For example, an infant might respond by following the gaze point of the parent looking at a target object. Although findings are somewhat mixed, it is believed that individuals with ASD are less likely to share attention with another person [7, 8], resulting in various socio-communicative challenges during development [6]. As a result, there is growing interest in identifying robust, reliable and valid biomarkers for determining differences in RJA behaviours between TD children and those with a diagnosis of ASD. The present study employed an eye-tracking paradigm during RJA tasks to examine and quantify differences between preschool children with ASD and a TD control group.

Recent advances in technology have allowed the quantification of different biological and behavioural markers that are useful in ASD research (see [9, 10] for a review). In particular, eye-tracking technology has been used to effectively distinguish ASD children from TD children [11]. In addition, it has been used to investigate differences in visual attention between children with and without a diagnosis of ASD (refer to [12–14] for a review). Furthermore, it has been used to quantify RJA behaviours and its construct validity has been established. For example, Navab, et al. [15] showed that eye-tracking measures, such as (1) the standard difference score defined as the number of participants’ first look at the distracter object subtracted from the number of participants’ first look towards the target object and (2) the percentage of accurate gaze shifts, during an RJA task were related to the Early Social Communication Scale (ESCS) distal pointing task where an examiner points at one of the three colourful posters hung on the testing room. Similarly, the accuracy of gaze shifts was found to be correlated with behavioural RJA on the ESCS [16]. Numerous eye-tracking measures have also been found to correlate with various clinical characteristics [17]. For example, the accuracy of gaze shifts correlates with Vineland Adaptive Behaviour Scales – Second Edition (VABS-II) Socialisation scores. [18] The present study investigated the construct validity of eye tracking (as it indexes RJA) and explored the associations between RJA behaviours quantified using eye-tracking and various clinical scores.

Studies that have compared eye tracking performance on RJA tasks across TD and ASD individuals have revealed mixed results, with some studies indicating a range of atypical responses in children with ASD [18–21] and others showing typical performance [22–26]. Eye-tracking studies investigating RJA behaviours in ASD individuals are summarised in Table 1. Variations in participant age, communicative content of the scene used, the inclusion of a face talking [27], emotional intensity [16], initiating direct gaze [28], visibility of the target [29], and the different nature of the stimuli (images or videos) [30] may all have contributed to differences between studies. While previous eye-tracking studies have been mostly consistent in finding that accuracy of gaze shifts is intact in infants with ASD or toddlers later diagnosed to have ASD [31], much less is known about RJA behaviours in older preschool children with ASD and TD children. Earlier research that used retrospective analysis of videos has observed that differences emerge later in development during preschool years [32, 33]. In this regard, accuracy in gaze shift has been found to be reduced in six-year-old children with ASD [18]. This finding has also been observed in adults with ASD [34]. Most RJA studies that included older participants typically have a wide age range. The present study recruited a cohort of ASD and TD participants from 31 to 73 months, the age range where diagnosis and assessment are typically performed [35].

**Table 1:** Eye-tracking studies exploring response to joint attention (RJA) behaviours in ASD individuals, sorted by age of participants

Article	Groups	Age	Nature of Stimuli	Main Results
Chawarska, et al. [19]	HR, LR	6 months	Dynamic stimuli of an actress, four toys and a table with ingredients for making sandwiches	6-month-old infants later diagnosed with ASD had less fixation duration to the social scene and face
Bedford, et al. [31]	HR, LR, ASD	6–10 months, 11–18 months	Dynamic stimuli where an actress moves her head to gaze at one of the two objects and fixates on that object	No group difference in gaze following accuracy, but at-risk infants with socio-communication difficulties ASD spent less time looking at the target object
Thorup, et al. [22]	HR, LR	10 months	Live interaction (not pre-recorded stimuli)	Infants in the HR group were more likely to follow gaze in the Eyes/Head condition than in the Eyes-Only condition
Nyström, et al. [39]	HR, LR	10 months	Live interaction with two conditions (Eyes/Head vs Eyes-Only condition)	No group differences in gaze following accuracy in Eyes/Head, Eyes-Only or combined conditions
Nyström, et al. [28]	HR, LR	10 months	Live interaction with an adult holding an object	Infants in the HR group spent less time looking at the stimuli during 300-1000 ms after bid of joint attention. Further analysis of the whole experiment revealed no significant group differences
Chawarska, et al. [20]	ASD, TD	13–25 months	Dynamic stimuli of an actress, four toys and a table with ingredients for making sandwiches	ASD children spent less time looking at stimuli and face. The decreased time spent looking at the scene was associated with increased symptom severity and lower non-verbal functioning. The decreased time spent looking at the face was associated with atypical language profiles
Franchini, et al. [16]	25 ASD, 21 TD	14–57 months	Dynamic stimuli of the same actress with varying intensity of facial expression in response to a moving object (1 out of 2 identical objects in the scene)	ASD participants were less responsive to bids of joint attention and spent less time looking at stimuli and face. Gaze accuracy differed depending on bids of joint attention
Parsons, et al. [26]	116 HR, 27 LR	15 months	Dynamic stimuli where an actress teaches word-object associations by shifting her head to an object and with varying exclamations	No group differences in gaze following accuracy, but children later diagnosed with ASD spent less time looking at either object. Attention distribution was correlated with both concurrent and later language abilities
Billeci, et al. [25]	17 ASD, 15 TD	18–30 months	Dynamic stimuli where an actress moves her head towards one of the two blocks and fixates on that block	No group differences in gaze following accuracy, number of transitions and fixation durations on objects in the scene
Gliga, et al. [40]	35 HR, 18 LR	36 months	Dynamic stimuli with a moving salient object and a stationary referent object. An actress says word-object associations and shifts her head to the referent object	No group differences observed in gaze following accuracy and amount of time looking at stimuli
Falck-Ytter, et al. [24]	13 ASD, 14 TD	34–60 months	Dynamic stimuli where an actress moves her head to gaze at one of the two objects and fixates on that object	No group differences observed in gaze following accuracy, but ASD children exhibit weaker processing bias for gazed-at objects
Vivanti, et al. [17]	35 ASD, 20 TD	48 months	Dynamic stimuli where an actress moves her head to gaze at one of the two objects and fixates on that object	ASD participants spent less time looking at the actor’s face and exhibited reduced gaze following when compared to TD participants. ADOS, VABS and MSEL items were found to be correlated with eye-tracking variables
**Current study**	60 ASD, 17 TD	31–73 months	Dynamic stimuli with two conditions (Eyes/Head vs Eyes-Only condition)	ASD participants were less like to follow gaze compared to TD participants in both conditions
Cilia, et al. [30]	28 ASD, 56 TD	31–154 months	Static and dynamic stimuli where an actor looks and/or verbalised or pointed and/or verbalised at a target	No group differences in time spent looking at the stimuli
Gillespie-Lynch, et al. [21]	21 ASD, 42 TD	28–80 months	Static stimuli for gaze cueing and dynamic stimuli for world learning task. Head and eye movements were used as a bid of joint attention	ASD participants exhibited reduced gaze following
Thorup, et al. [41]	16 ASD, 17 TD	38–112 months	Dynamic stimuli where an actress is seated behind a table with four objects. The study tested whether objects perceived as highly interesting by ASD children affect gaze following behaviours	No group differences in gaze following accuracy. ASD participants exhibited lower first fixation durations in the baseline case where ordinary objects were used
Congiu, et al. [29]	25 ASD, 25 TD	45–103 months	Dynamic stimuli where an actress showed an object, hid the object under one of two identical cups, shuffled the cups, looked at the camera and gazed towards the cup that contains the object. The study contained two conditions: (1) perceptual and (2) representational (where the shuffling was not shown)	ASD participants were less accurate in following gaze and spent less time looking at the gaze-at object during representational condition
Falck-Ytter, et al. [18]	40 ASD, 21 TD	72 months	Dynamic stimuli where an actress gazed, pointed or did both at one out of three objects	ASD participants were less accurate in following gaze. Difference score (DS) and gaze accuracy were positively correlated with VABS-II communication scores. DS was also positively correlated with VABS-II socialisation scores. ASD participants were slower to look at the gaze-at object
Swanson and Siller [42]	21 ASD, 24 TD	84 months	Dynamic stimuli where an actress moved her head and gazed at one of the four corners of the screen. There were two conditions: (1) congruent condition where the actress looked at a corner where the object is located and (2) incongruent condition where the actress looked at a corner where the object is not located	TD participants spent longer first fixation duration to the target object in the congruent condition than in the incongruent condition. ASD participants viewed the targets in an indistinguishable manner in both conditions. ASD children who scored high in SRS Social Awareness subscale failed to follow gaze
Griffin and Scherf [43]	35 ASD, 35 TD	10–18 years old	Dynamic stimuli where an actress directed eye gaze to a single target object in a complex scene	ASD participants exhibited difficulties in their ability to correctly follow eye gaze to identify gazed-at objects. No group differences in the amount of time spent looking at faces and gazed-at objects
Riby, et al. [44]	ASD, TD	11 years old	Static stimuli where a person’s gaze was directed to a target object in a complex scene. The study includes two conditions: (1) participants were initially told to look at the pictures then (2) asked to identify the gazed-at object	In both conditions, ASD participants recorded less fixation duration to faces, eyes and gazed-at objects. In the second condition, ASD participants looked more at the face and eyes but did not follow the gaze direction. Higher functioning ASD individuals scored more accurately in identifying the gazed-at object
Caruana, et al. [34]	17 ASD, 17 TD	26 years old	Dynamic stimuli where participants played a cooperative game with a human face avatar that cues for the location of the target using eye gaze or displaying an arrow	In the eye gaze condition, ASD adults were less accurate at responding to joint attention and initially slower to respond but their performance improved significantly. This indicates that ASD adults have difficulties responding to gaze cues

*HR* High Risk, *LR* Low Risk, *ADOS* Autism Diagnostic Observation Schedule, *ASD* Autism Spectrum Disorder, *TD* Typical Development, *VABS* Vineland Adaptive Behaviour Scales, *MSEL* Mullen Scale of Early Learning, *SRS* Social Responsiveness Scale

Various visual stimuli and experimental paradigms have been used in eye-tracking research with children on the spectrum [36]. For example, some eye-tracking studies have used static or dynamic stimuli that had an actor turning his/her head to initiate joint attention, possibly eliminating any confounding effect of either eye gaze or head movement on the ability of participants to respond to a bid for joint attention. Using retrospective video analysis, Presmanes, et al. [37] studied the effects of different attentional cues on RJA and found that there was no difference in the accuracy of gaze shifts between younger siblings of children with ASD and infants in the control group, when combinations of verbal and non-verbal cues were used simultaneously. However, lower accuracy of gaze shifts was found in the younger siblings of ASD children when fewer cues were presented. A previous study showed that gaze following performance increased when pointing with language cues was added [38]. The effect of head movement on the ability of infants to follow gaze has also been recently studied using eye-tracking during a live interaction [22, 39]. One study showed that infants at familial risk for ASD were less likely to follow gaze with the Eyes-Only condition when compared to the Eyes/Head condition [22]. This is contrary to neurotypical infants, whose accuracy of gaze shifts did not vary between those two conditions. While more naturalistic stimuli have been shown to evoke different neural and behavioural responses than pre-recorded stimuli, the former offers poorer experimental control. The present study investigated whether the results seen in naturalistic studies also hold in preschool children during an experimental eye-tracking paradigm.

The present study aimed to identify differences in RJA behaviours between ASD and TD preschool children, building on previous research findings [24, 40]. Two different conditions (Head and Eyes vs. Eyes-Only condition) were therefore included using pre-recorded stimuli to compare RJA behaviours between ASD and TD children. Further correlations between eye-tracking measures and various clinical scores were examined. We hypothesised that this study would independently replicate and extend previous findings that identified differences in RJA behaviours between ASD and TD children. Specifically, we hypothesised that ASD participants would have reduced RJA behaviours when eye gaze alone is used to initiate joint attention, a finding that was observed previously during live interactions with ASD infants [22]. A further hypothesis was that eye-tracking measures would be meaningfully correlated with clinical information, suggesting that eye tracking could be used as a helpful biomarker in clinical assessments of ASD.

## Methods

### Participants

Participants were children between 31 months and 6 years of age with a confirmed ASD diagnosis (*N* = 60; 51 males) and TD children (*N* = 17; 8 males). Thirty-four children (29 males) in the ASD group were recruited from the KU Marcia Burgess Autism Specific Early Learning and Care Centre (ASELCC) and 26 children (22 males) were recruited from a Child Development Unit (CDU) at the Children’s Hospital in Westmead, New South Wales, Australia. TD children were recruited from the KU Children’s Services (CS) preschool in Liverpool, New South Wales, Australia. All ASD participants met the criteria for ASD based on the Diagnostic and Statistical Manual of Mental Disorders (DSM-5) criteria [1] for ASD and the diagnosis was confirmed using the Autism Diagnostic Observation Schedule – Second Edition (ADOS-2) [45]. No specific exclusion criteria were applied for ASD participants. Participants with known neurodevelopmental disorders, significant developmental delays and reported visual/hearing difficulties were excluded from participation in the TD group. No child had any visual acuity problems. This study was approved by the University of New South Wales Human Research Ethics Committee (HC14267). Written informed consent was obtained from the participants’ parents/legally authorised representatives. All methods were carried out in accordance with relevant guidelines and regulations.

### Clinical measures

Participants were administered a battery of clinical and behavioural assessments for determining autism symptomatology, developmental skills, and adaptive functioning. These assessments were conducted on-site, either at ASELCC, CS or CDU. The assessments were completed in approximately 2.5 h per participant. This was done as part of a clinical assessment or the intake assessment for entry to an early intervention program and was at times done spread over 2 or 3 sittings or with smaller breaks depending on the capacity of the child.

The Autism Diagnostic Observation Schedule, Second Edition (ADOS-2) is a semi-structured standardised assessment instrument for ASD diagnosis in individuals aged 12 months to adulthood [45]. It is used to quantify autism symptomatology across social interaction, communication, play, repetitive behaviours and imaginative use of materials. Depending on the participant’s age and language ability, the appropriate module of the ADOS-2 was administered by qualified research staff (Module 1: *N* = 41, Module 2: *N* = 8, Module 3: *N* = 11). Higher ADOS scores are indicative of a greater degree of ASD symptomatology.

The Social Communication Questionnaire (SCQ) is a parent-reported screening tool used to quantify autism-specific symptoms [46]. It consists of 40 items and yields a total score and three subscale scores in different domains: Communication, Social Interaction and Restricted Repetitive Behaviour.

The Mullen Scale of Early Learning (MSEL) is a standardised measure of cognitive and motor development [47]. It provides an estimate of verbal and non-verbal abilities of children less than 6 years of age. It yields standardised T Scores, age equivalent scores and raw scores on different subscales: Visual Reception, Fine Motor, Gross Motor, Receptive Language and Expressive Language. Since the Gross Motor subscale was not administered in this study, age equivalent (AE) scores and developmental quotient (DQ) scores on the remaining subscales were used for analysis. DQ scores were calculated by dividing the age equivalent scores by the chronological age and the result was multiplied by 100. Verbal DQ is the average value of the receptive language DQ score and the expressive language DQ score. On the other hand, non-verbal DQ is the average value of the visual reception and fine motor DQ scores. As a result, possible effects of chronological and developmental age are explored. In addition, both verbal and non-verbal DQs were used as a proxy for a measure of intelligence quotient (IQ).

The Vineland Adaptive Behaviour Scales – Second Edition (VABS-II) is a well-established and reliable parent-reported measure of the child’s daily adaptive functioning [48]. It yields an overall composite score and subscale standard scores in the following domains, including Communication, Daily Living Skills, Socialisation, and Motor Skills. All subscale standard scores were used for analysis.

### Eye tracking

#### Apparatus and procedure

Eye-tracking data was collected using the Tobii X2-60 eye tracker and analysed using Tobii Studio software [49]. Each participant entered a quiet room and sat approximately 60 cm in front of a 22″ widescreen monitor with a resolution of 1680 × 1050 pixels. Novel stimuli for assessing response to joint attention were shown at the location of recruitment and took approximately five minutes to finish. To ensure accurate eye tracking, a built-in five-point calibration procedure in Tobii Studio was completed for each participant before administering the task. The calibration procedure required gaze following on an image of an animal paired with auditory cues, starting with the centre of the screen, and moving across the four corners of the screen.

#### Response to joint attention

**Fig. 1:**
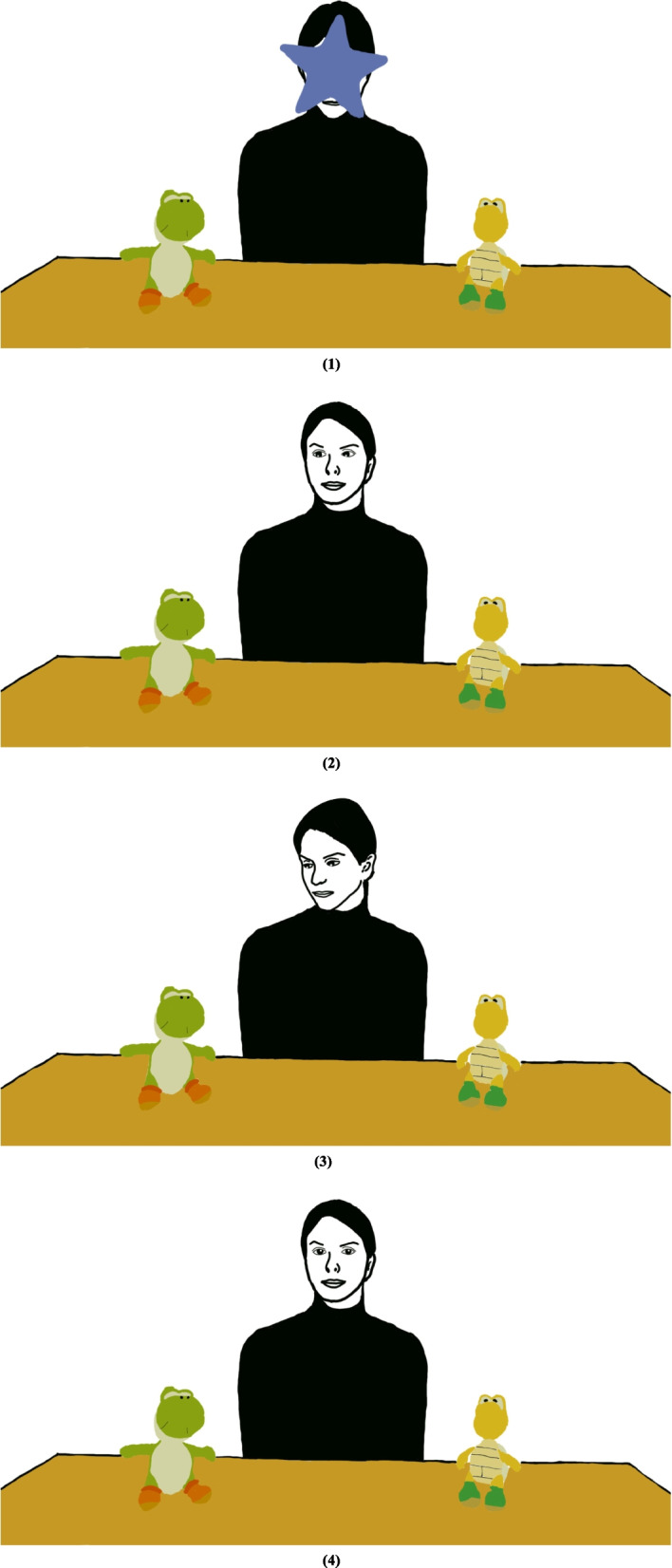
Stylised representation of the eye-tracking task, described based on the row number: 1.) an animated attention-getter (star) accompanied by a sound covers the actor’s face, attracting the child’s attention. 2.) the actor initiates joint attention using the Eyes-Only condition. 3.) the actor initiates joint attention using the Head/Eyes condition. 4.) the actor shifts her gaze back to the camera

Eight videos were presented of a female actor seated behind a table on which two toys were placed, one to each side of the actor. The task was similar to previous studies [15, 18, 24–26, 31, 41, 50–52] with additional conditions controlling for the actor’s initiation of joint attention (Eyes-Only condition or Head/Eyes condition). Each video consisted of multiple phases (refer to Fig. 1). In the first phase, an animated attention-getter (star) accompanied by a sound covers the actor’s face, attracting the child’s attention. This phase lasted 3.0 s. In the second phase, the animation disappears, and the actor looks directly at the camera and smiles for 3.0 s, engaging the child’s attention. Afterwards, the actor initiates joint attention for 4.0 s by either (a) shifting and holding her eye gaze towards one of the toys (Eyes-Only condition) or (b) shifting and holding her gaze and simultaneously turning her head towards one of the toys (Head/Eyes condition). Finally, the actor shifts her gaze back to the camera for 2.0 s. For each condition, there were four visually similar designs, counterbalanced for placement of the target toy (left or right) to ensure that there was a minimal influence of the participant’s looking preference. As a result, there was a total of 8 trials with four blocks. Each block had two trials where the target object's location was counterbalanced. The experiment started with one block of the same condition followed by another block of the other condition. The experiment finished when 8 videos were displayed. The presentation of conditions was counterbalanced, so half of the participants started the experiment with the Head/Eyes condition while the other half started with the Eyes-Only condition.

#### Eye-tracking measures

Five different eye-tracking measures were adopted from previous studies [15, 50, 53, 54].

The standard difference score was computed by subtracting the frequency with which the participant first looks from the actor to the distracter object from the frequency with which the first look was towards the target object [15, 53, 54]. This is similar to the eye-tracking measure used during a live interaction with the same experimental conditions (Head/Eyes and Eyes-Only conditions) [39].

The percentage of accurate gaze shifts was computed by dividing the number of correct trials (trials where the participant looks towards the same toy that the actor is directing their attention towards) by the total number of valid trials [15, 53].

The restrained standard difference score (RSDS) was computed by dividing the standard difference score by the total number of trials in which the participant looked at either the target or distracter object [15, 50].

The restrained duration difference score (RDDS) was computed by dividing the difference of the total duration (in milliseconds) of all fixations upon the distracter object from the total duration of all fixations upon the target object by the total duration of all fixations upon either object [15, 50].

The response time (RT) was computed by measuring the number of milliseconds between the presentation of the joint attention cue and the onset of the participant’s fixation on the correct target location [53].

#### Data processing and statistical analysis

Fixations and saccades were identified using the identification velocity threshold (IV-T) filter [55] in Tobii Studio. For the purpose of exclusion criteria, all trials were divided temporally into the four phases described above. Three different areas of interest (AOIs) within each video were defined around the face, target toy and distracter toy. Python scripts were written to extract gaze information around these AOIs in the four phases. Trials were excluded if there was no fixation recorded on the face AOI during the attention-getter and/or the smiling phase, as this indicated that the child was not reliably participating in the task. To be included in the analysis, each participant required at least 1 valid trial (25%) per condition. This resulted in 77 infants being included in the Eyes/Head and Eyes-Only condition comparison, with an average of 6.68 valid trials per participant.

Repeated-measures analysis of variance (ANOVA) was used to investigate differences in eye-tracking across the two groups and the two experimental conditions. Specifically, each eye-tracking dependent variable (gaze, SDS, RSDS, RDDS, RT) was investigated using a 2 × 2 ANOVA with group (ASD vs TD) as a between-subjects variable, and condition (Eyes-Only vs Eyes/Head) as a repeated measures variable. Each ANOVA was subject to a Family-wise error rate of 0.05. In addition, all post hoc analyses were subject to Bonferroni corrections in order to reduce the risk of Type I errors. A separate analysis including age as a covariate was also performed. In addition, another analysis was conducted with a restricted age range. Effect sizes were estimated by partial eta squared (η^2^; values from 0.01 to 0.06 are considered a small effect, values from 0.06 and 0.14 are considered a medium effect and values above 0.14 are considered a large effect) [25, 56]. Correlations between each eye-tracking measure and each clinical measure were assessed using Spearman’s correlations.

All statistical analysis was performed in IBM SPSS Statistics Version 26.

## Results

The demographic information of the participants is shown in Table 2. Children with ASD had a mean age of 4.57 (0.82) years while TD participants had a mean age of 4.61 (0.47) years. There was no significant difference in age between the two groups, F(1,75) = 8.199, *p* = 0.833. The gender distribution was significantly different between groups with a higher proportion of males in the ASD group, X^2^(1) = 10.646, *p* = 0.001. However, performance did not differ between boys and girls in either the Eyes/Head condition, F(1,69) = -0.855, *p* = 0.395, or the Eyes-Only condition, F(1,69) = -0.836, *p* = 0.406, across both groups. Bayesian statistics (Independent Samples Normal) analysis was also conducted to confirm this. The most likely difference between the mean gaze accuracy of boys and girls in the ASD group were -0.669 and -0.0567 in the Eyes/Head and Eyes-Only conditions respectively. However, the Bayesian factors (BF) were 3.031 and 3.116 respectively, suggesting the difference in mean performance between boys and girls in the ASD group is not statistically significant. Similarly, the difference in mean performance between boys and girls in the TD group was not significant in either condition (Eyes/Head condition: mean difference in gaze accuracy: -0.0382, BF = 2.882; Eyes-Only condition: mean difference of gaze accuracy:1.609, BF = 1.806).

**Table 2:** Participant demographic information

Measure	ASD	TD	Test statistic (t or X^2^)	*p*-value
Age (years)	4.57 (0.82), *N* = 60	4.61 (0.47), N = 17	8.199	0.833
Sex			10.646	0.001**
Male	51	8		
Female	9	9		
Autism Diagnostic Observation Schedule (ADOS)				
Social Affect	13.07 (3.29), *N* = 60			
Restricted Repetitive Behaviour	4.73 (1.74), *N* = 60			
Calibrated Severity Score	7.33 (1.14), *N* = 60			
Vineland Adaptive Behaviour Scales—(VABS-II)				
Communication, Standard Score (SS)	69.69 (19.07), *N* = 52			
Daily living, SS	69.38 (16.86), *N* = 52			
Socialization, SS	67.27 (13.02), *N* = 52			
Motor Skills, SS	76.08 (14.66), *N* = 52			
Mullen Scales of Early Learning (MSEL)				
Visual reception, Age Equivalent (AE)	30.10 (17.97), *N* = 52	53.88 (8.62), *N* = 17	5.25	< 0.001**
Fine motor, AE	30.5 (13.09), *N* = 52	53.47 (9.44), *N* = 17	6.68	< 0.001**
Receptive language, AE	25.06 (16.72), *N* = 52	46.59 (9.62), *N* = 17	5.03	< 0.001**
Expressive language, AE	25.10 (14.39), *N* = 52	43.65 (7.92), *N* = 17	5.05	< 0.001**
Verbal Developmental Quotient	45.53 (24.82), N = 51	82.08 (15.85), N = 17	5.68	<0.001**
Non-Verbal Developmental Quotient	55.69 (25.24), N = 51	97.72 (17.27), N = 17	6.37	<0.001**
Social Communication Questionnaire (SCQ)	0.54 (0.17), *N* = 41	0.17 (0.11), *N* = 16	-8.04	< 0.001**

^**^Indicates *p*-values < 0.001

There were significant differences in cognitive level as determined using the MSEL and autism features as measured using the SCQ scores between the ASD and TD groups. Because parents did not complete every questionnaire, the sample contained missing data. Missing data were dealt with by case-wise exclusion. The total number of valid trials did not differ between the two groups, for either the Eyes and Head condition (ASD: 3.42(0.70), TD: 3.53(0.72), *p* = 0.56), the Eyes-Only condition (ASD: 3.42(0.72), TD: 3.56(0.51), *p* = 0.45), or when data from both conditions were combined (ASD: 6.55 (1.57), TD: 7.19 (0.98), *p* = 0.13). This suggests that there was no significant difference in the amount of valid data available in the two groups.

A calibration quality assessment was performed to rule out the possibility of eye-tracking data quality as a confounding factor. In this assessment, a toy accompanied by a sound was used to attract the participants’ gaze to the calibration point in the middle of the screen. The mean distance between the detected fixation locations and the calibration point was calculated as a measure of accuracy. A t-test between the groups showed no significant difference between the groups, suggesting that data quality did not differ between the two groups: t(57) = 0.334, *p* = 0.739, ASD: 46.49 pixels (23.87), TD: 48.76 pixels (19.00).

An additional data quality assessment was performed to determine the overall nature of the visual attention of the participants in both conditions. In particular, the average amount of time spent looking at the stimuli in each condition was computed. There was a significant main effect of condition, F(1,69) = 6.256, *p* = 0.015. On average, participants spent around 35 s longer in the Eyes/Head condition (6.26(2.47)) than in the Eyes-Only condition (5.68(2.91)). There was no significant main effect of group, F(1,69) = 3.718, *p* = 0.058, and no significant interaction effect, F(1,69) = 0.00, *p* = 0.991. These analyses of quality suggest that it is unlikely that differences in data quality and general attention were responsible for clinically meaningful group differences in RJA.

### Standard difference score (SDS)

**Fig. 2:**
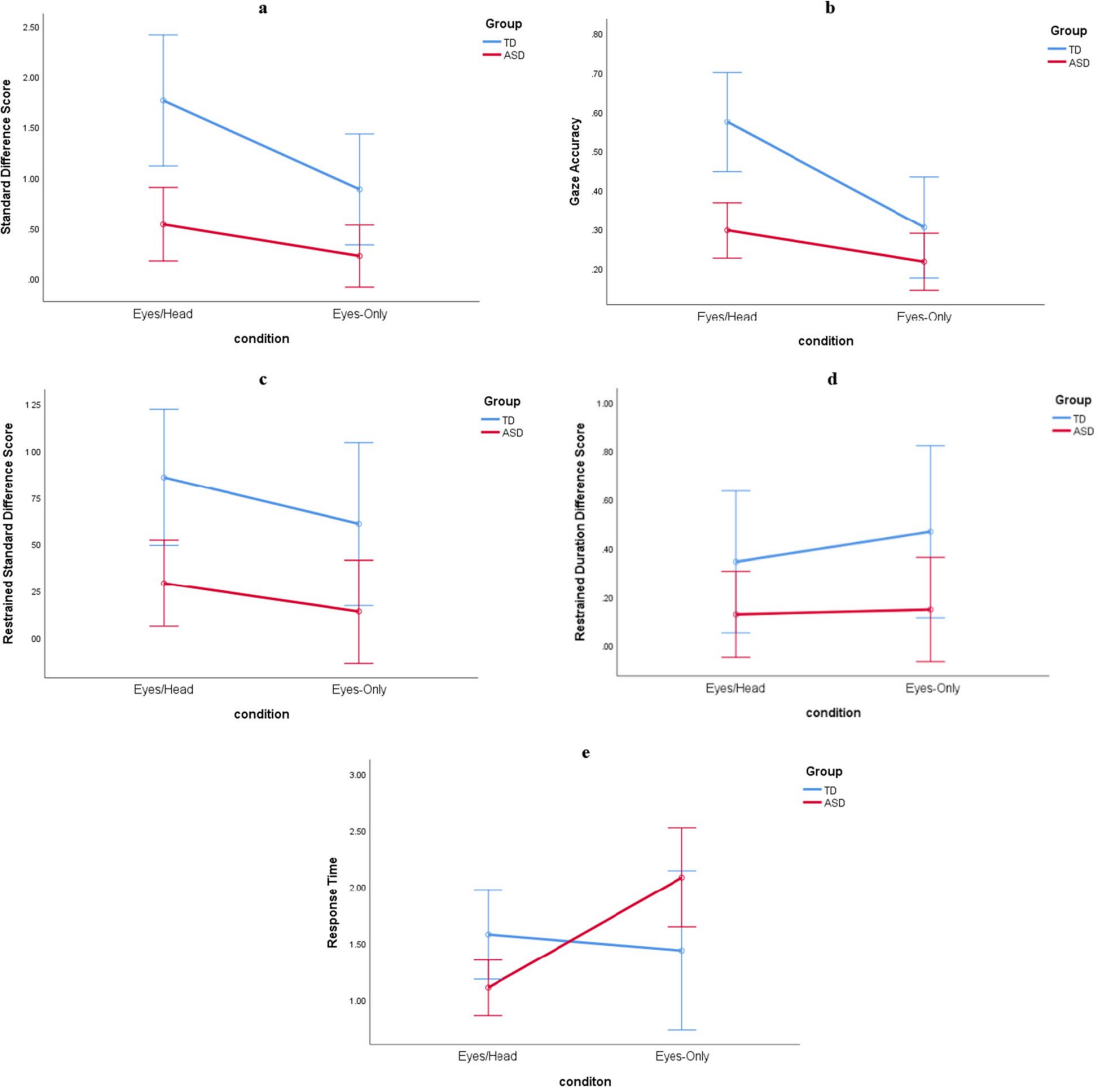
Different eye-tracking measures of ASD and TD participants in two conditions. Error bars show 95% CI

Standard difference score (SDS) refers to the number of participants’ first look at the distracter object subtracted from the number of participants’ first look towards the target object. Hence, a positive (or higher) SDS means that the participant responded to the joint attention cue more frequently (indicative of better joint attention). The ANOVA investigating standard difference scores revealed a main effect of group (as seen in Fig. 2a), with TD children achieving higher SDS, F(1,69) = 11.205, *p* = 0.001, η^2^ = 0.140. There was also a main effect of condition, as the performance was better in the Eye/Head condition than in the Eyes-Only condition, F(1,69) = 8.916, *p* = 0.004, η^2^ = 0.114. There was no interaction effect between group and condition, F(1,69) = 2.004, *p* = 0.161, η^2^ = 0.028. When age was included as a covariate, the groups still differed significantly in terms of SDS, as the TD group reported higher SDS, F(1,68) = 11.272, *p* = 0.001, η^2^ = 0.142. There was no significant effect of condition, F(1,68) = 1.399, *p* = 0.241, η^2^ = 0.020, no interaction between condition and age, F(1,68) = 0.401, *p* = 0.529, η^2^ = 0.006, and no interaction between condition and participant group, F(1,68) = 1.987, *p* = 0.163, η^2^ = 0.028.

### Accuracy of gaze shifts

The accuracy of gaze shifts was computed by dividing the number of correct trials by the total number of valid trials. The groups significantly differed in terms of the accuracy of gaze shifts (see Fig. 2b), with the TD group demonstrating higher accuracy, F(1,69) = 9.870, *p* = 0.002, η^2^ = 0.125. There was a main effect of condition, as accuracy was increased for the Eyes/Head condition, F(1,69) = 14.990, *p* < 0.00002, η^2^ = 0.178. There was also a significant interaction effect between condition and group, F(1,69) = 4.391, *p* = 0.040, η^2^ = 0.060. Specifically, post hoc, Bonferroni-corrected contrasts showed that gaze accuracy was significantly reduced for the Eyes-Only condition (mean: 0.30, std: 0.29) compared with the Eyes/Head condition (mean:0.57, std: 0.21), for TD participants only, t(16) = 3.922, *p* = 0.001; critical alpha = 0.025. By contrast, there was no significant difference between the Eyes Only (mean:0.22, std: 0.26) and Eyes/Head conditions (mean:0.30, std: 0.28) for the ASD group, t(53) = 1.753, *p* = 0.085 (critical alpha = 0.025 given the Bonferroni correction). Importantly, even when adjusted for age, the groups still differed significantly in terms of the accuracy of gaze shifts, F(1,68) = 9.888, *p* = 0.002, η^2^ = 0.127. The significant interaction effect between condition and participant group also remained, as described above, F(1,68) = 4.328, *p* = 0.041, η^2^ = 0.060. However, there was no significant effect of condition, F(1,68) = 0.548, *p* = 0.462, η^2^ = 0.008, and no interaction between condition and age, F(1,68) = 0.000, *p* = 0.988, η^2^ = 0.000.

### Restrained standard difference score (RSDS)

RSDS was computed by dividing the standard difference score by the total number of trials in which the participant looked at either the target or distracter object. RSDS showed a significant effect of group (see Fig. 2c), with RSDS significantly higher for the TD group, F(1,47) = 7.287, *p* = 0.010, η^2^ = 0.134. There was no main effect of condition, F(1,47) = 2.117, *p* = 1.52, η^2^ = 0.043, and no interaction effect between group and condition, F(1,47) = 0.125, *p* = 0.726, η^2^ = 0.003. When adjusted for age, the groups continued to differ in terms of RSDS, F(1,46) = 7.115, *p* = 0.011, η^2^ = 0.134. There was no significant effect of condition, F(1,46) = 0.921, *p* = 0.342, η^2^ = 0.020, no interaction between condition and age, F(1,46) = 0.524, *p* = 0.473, η^2^ = 0.011, and no interaction effect between condition and participant group, F(1,46) = 0.142, *p* = 0.708, η^2^ = 0.003.

### Restrained duration difference score (RDDS)

RDDS was computed by dividing the difference in the total duration (in milliseconds) of all fixations on the distracter object from the total duration of all fixations on the target object by the total duration of all fixations upon either object. Hence, positive (or higher) RDDS means that the participant allocated more attention to the target object than the distracter object. There was no main effect of condition (see Fig. 2d), F(1,54) = 0.322, *p* = 0.573, η^2^ = 0.006, and no interaction effect between group and condition, F(1,54) = 0.168, *p* = 0.684, η^2^ = 0.003. However, the effect of group on RDDS trended towards significance, F(1,54) = 3.612, *p* = 0.063, η^2^ = 0.063, suggesting that TD participants allocated more attention to the target than the distracter when compared to ASD participants. When adjusted for age, there were no significant main or interaction effects (highest F = 3.974, *p* = 0.051, η^2^ = 0.070).

### Response times (RT)

Response Times (RT) measures the speed with which participants were able to correctly look at the target object after responding to a joint attention bid. There was no main effect of condition (refer to Fig. 2e), F(1,45) = 3.093, *p* = 0.085, η^2^ = 0.064, and no main effect of group, F(1,45) = 0.139, *p* = 0.711, η^2^ = 0.003. However, there was a significant interaction effect between group and condition, F(1,45) = 5.564, *p* = 0.023, η^2^ = 0.110. Post-hoc, Bonferroni-corrected contrasts revealed that ASD participants’ response times were slower in the Eyes-Only condition (mean RT = 2.08, std = 1.35) compared to the Eyes/Head condition (mean RT = 1.11, std = 0.78) (t(33) = -3.769, *p* = 0.001; critical alpha = 0.025). On the other hand, RT did not differ across conditions for the TD participant group, t(12) = 0.399, *p* = 0.697: Eyes/Head: mean RT = 1.58, std = 0.48 and Eyes-Only: mean RT: 1.43, std = 1.00. These results suggest that ASD children were slower to allocate attention to the target object when only eye gaze information was available compared with when there was a movement of both eye and head directly towards the target object.

### Age-restricted analysis

Given that the age range was considerably broader for the ASD group compared to the TD group, all of the above analyses were re-run with a reduced sample of ASD children (*N* = 33), which acted to match age across the two groups. Specifically, ASD children were excluded from the analysis if their age was not within the age range of the group of TD children (3.96 – 5.41 years). Importantly, the results reported above did not change in terms of statistical significance when the analysis was restricted to this smaller group of participants, thereby demonstrating that our primary results were not driven by the inclusion of a wider age range of ASD participants.

### Correlations between eye-tracking measures and clinical information

Correlations between eye-tracking measures and clinical information are shown in Table 3 for the ASD group. The analysis relating RJA eye-tracking variables to different clinical scores showed numerous significant associations for ASD participants. SDS was significantly positively correlated with Visual Reception, Fine Motor, Receptive Language and Expressive Language on MSEL, as well as Communication, Daily Living Skills and Socialisation on VABS. SDS was negatively correlated with Calibrated Severity Score and Social Affect on ADOS as well as SCQ scores.

**Table 3:** Spearman’s correlations between eye-tracking variables and clinical characteristics in the ASD group. Correlation coefficients (with corresponding *p*-values) are listed

Eye-tracking variables					
Clinical Information	SDS	Accuracy of Gaze Shifts	RSDS	RDDS	RT
**ADOS**					
Calibrated Severity Score	**-0.303 (0.019)**	**-0.282 (0.029)**	**-0.292 (0.029)**	**-0.357 (0.005)**	-0.152(0.357)
Social Affect	-0.415 (0.001)*	-0.451 (< 0.001)*	-0.414 (0.002)*	**-0.351 (0.006)**	-0.360(0.830)
Restricted Repetitive Behaviour	-0.222 (0.088)	-0.246 (0.058)	-0.148 (0.275)	**-0.272 (0.036)**	-0.227(0.164)
**MSEL**					
Visual Reception, AE	**0.370 (0.007)**	**0.373 (0.006)**	**0.326 (0.024)**	**0.337 (0.015)**	0.053(0.772)
Fine Motor, AE	**0.365 (0.008)**	**0.381 (0.005)**	**0.348 (0.016)**	**0.379 (0.006)**	-0.002(0.990)
Receptive Language, AE	0.470 (< 0.001)*	0.452 (0.001)*	0.436 (0.002)*	0.422 (0.002)*	0.061(0.740)
Expressive Language, AE	0.441 (0.001)*	0.465 (0.001)*	**0.412 (0.004)**	**0.385 (0.005)**	-0.021(0.909)
Verbal DQ	0.472 (< 0.001)*	0.492 (< 0.001)*	0.432 (0.002)*	**0.341 (0.014)**	-0.057 (0.729)
Non-verbal DQ	**0.296 (0.035)**	**0.318 (0.023)**	0.215 (0.143)	0.222 (0.118)	-0.021(0.899)
**VABS**					
Communication, SS	0.445 (0.001)*	0.497 (0.000)*	**0.297 (0.043)**	0.211 (0.137)	0.133(0.469)
Daily Living Skills, SS	**0.391 (0.004)**	**0.386 (0.005)**	0.264 (0.072)	0.203 (0.152)	0.032(0.861)
Socialisation, SS	0.424 (0.002)*	0.448 (0.001)*	**0.320 (0.028)**	0.227 (0.110)	0.242(0.183)
Motor Skills, SS	0.205 (0.146)	0.209 (0.138)	0.102 (0.495)	0.170 (0.233)	0.015(0.933)
**SCQ**	**-0.361 (0.028)**	**-0.393 (0.016)**	-0.251 (0.146)	-0.054 (0.746)	0.025(0.913)

*ADOS* Autism Diagnostic Observation Schedule, *MSEL* Mullen Scale of Early Learning, *VABS* Vineland Adaptive Behaviour Scales, *SCQ* Social Communication Questionnaire, *SDS* Standard Difference Score, *RSDS* Restrained Standard Difference Score, *RDDS* Restrained Duration Difference Score, *RT* Response Time

Text in bold indicates *p*-values <0.05. ^*^Indicates *p*-values that would be significant at the 0.002 level

Accuracy of gaze shifts was found to be correlated to Visual Reception, Fine Motor, Receptive Language, Expressive Language on MSEL and Communication, Daily Living Skills and Socialisation on VABS. Accuracy of gaze shifts was found to be negatively correlated to Calibrated Severity Score and Social Affect on ADOS as well as SCQ scores. RSDS was found to be correlated to Visual Reception, Fine Motor, Receptive Language, Expressive Language on MSEL and Communication and Socialisation on VABS. RSDS and RDDS were found to be negatively correlated to Calibrated Severity Score and Social Affect on ADOS. RDDS was found to be correlated to Visual Reception, Fine Motor, Receptive Language, and Expressive Language on MSEL. RDDS was also found to be negatively correlated to Restricted Repetitive Behaviours on ADOS.

All of the above analyses were re-run with a reduced sample of children from the ASD group (*N* = 33) to ensure that the age ranges were matched across the two groups (specifically the acceptable age was restricted to 3.96 – 5.41 years). Table 5 shows Spearman’s correlations between eye-tracking measures and clinical information using the age-restricted sample. Most correlations in MSEL and VABS domains were retained even after controlling for the age of the ASD group. Interestingly, the Response time (RT) was found to be negatively correlated with the ADOS severity score after controlling for age. On the other hand, no correlations between eye-tracking measures and clinical characteristics were significant for the TD group (refer to Table 4).

**Table 4:** Spearman’s correlations between eye-tracking variables and clinical information in the TD group. Correlation coefficients (with corresponding *p*-values) are reported

Eye-tracking variables					
Clinical Information	SDS	Accuracy of Gaze Shifts	RSDS	RDDS	RT
**MSEL**					
Visual Reception, AE	-0.059 (0.821)	-0.135 (0.605)	-0.143 (0.584)	-0.337 (0.186)	0.082 (0.754)
Fine Motor, AE	-0.093 (0.724)	-0.220 (0.397)	-0.222 (0.391)	-0.372 (0.142)	0.064 (0.807)
Receptive Language, AE	-0.148 (0.570)	-0.142 (0.587)	-0.090 (0.731)	-0.247 (0.338)	-0.129 (0.621)
Expressive Language, AE	-0.368 (0.146)	-0.252 (0.329)	-0.288 (0.263)	-0.202 (0.436)	-0.347 (0.173)
Verbal DQ	-0.378 (0.134)	-0.367 (0.147)	-0.379 (0.134)	-0.064 (0.808)	-0.350 (0.168)
Non-verbal DQ	-0.248 (0.338)	-0.360 (0.156)	-0.348 (0.171)	-0.252 (0.328)	0.017 (0.948)
**SCQ**	-0.011 (0.969)	-0.105 (0.698)	0.430 (0.096)	0.148 (0.583)	0.214 (0.427)

*ADOS* Autism Diagnostic Observation Schedule, *MSEL* Mullen Scale of Early Learning, *VABS* Vineland Adaptive Behaviour Scales, *SCQ* Social Communication Questionnaire, *SDS* Standard Difference Score, *RSDS* Restrained Standard Difference Score, *RDDS* Restrained Duration Difference Score, *RT* Response Time

To control for the multiple comparisons, Tables 3, 4 and 5 also demonstrate the correlations that remain statistically significant when a much more conservative critical alpha of 0.002 is applied (Bonferroni-corrected critical alpha, to account for at least 14 correlations being computed for each dependent variable). The results suggest that more advanced cognitive and language skills (as measured by the MSEL) were associated with better joint attention skills (as measured by all the eye-tracking measures) in children with ASD. Initial gaze location and accurate gaze location were both positively correlated with adaptive functioning, as measured via the VABS-II. There was no correlation between eye gaze profile and scores in the motor skills domain. Collectively, these findings indicate that more accurate eye-tracking gaze profiles were associated with better early learning and more adaptive functioning. Furthermore, given that Social Communication Questionnaire scores were negatively correlated with eye-tracking measures in children with ASD, suggesting that more severe ASD symptomatology is associated with worse gaze profiles. There were no correlations for the TD group, as shown in Table 4. The correlations for the restricted-age ASD group in Table 5 show similar trends as for the full ASD group in Table 3.

**Table 5:** Spearman’s correlations between eye-tracking variables and clinical characteristics in the age-restricted ASD group. Correlation coefficients (with corresponding *p*-values) are reported

Eye-tracking variables					
Clinical Information	SDS	Accuracy of Gaze Shifts	RSDS	RDDS	RT
**ADOS**					
Calibrated Severity Score	-0.265 (0.136)	-0.318 (0.072)	-0.154 (0.401)	-0.244 (0.170)	**-0.382 (0.037)**
Social Affect	-0.323 (0.067)	**-0.425 (0.014)**	-0.259 (0.152)	-0.225 (0.208)	0.004 (0.982)
Restricted Repetitive Behaviour	-0.24 (0.179)	-0.338 (0.055)	-0.054 (0.769)	-0.148 (0.411)	-0.333 (0.072)
**MSEL**					
Visual Reception, AE	**0.426 (0.021)**	**0.471 (0.010)**	**0.384 (0.044)**	0.301 (0.113)	0.015 (0.943)
Fine Motor, AE	0.36 (0.055)	**0.434 (0.019)**	0.307 (0.112)	0.244 (0.203)	0.067 (0.747)
Receptive Language, AE	**0.54 (0.003)**	0.597 (0.001)*	**0.537 (0.003)**	**0.468 (0.010)**	-0.034 (0.868)
Expressive Language, AE	**0.534 (0.003)**	0.621 (< 0.001)*	**0.517 (0.005)**	**0.371 (0.048)**	0.041 (0.843)
Verbal DQ	**0.506 (0.005)**	0.588 (0.001)*	**0.482 (0.009)**	0.342 (0.070)	-0.026 (0.901)
Non-verbal DQ	**0.378 (0.043)**	**0.427 (0.021)**	0.312 (0.106)	0.208 (0.278)	-0.022 (0.914)
**VABS**					
Communication, SS	**0.563 (0.003)**	0.655 (< 0.001)*	**0.453 (0.023)**	0.307 (0.127)	0.057 (0.796)
Daily Living Skills, SS	**0.424 (0.031)**	**0.500 (0.009)**	0.358 (0.079)	0.298 (0.139)	0.038 (0.865)
Socialisation, SS	**0.421 (0.032)**	**0.431 (0.028)**	0.310 (0.132)	0.179 (0.381)	0.146 (0.507)
Motor Skills, SS	0.244 (0.229)	0.296 (0.142)	0.208 (0.320)	0.203 (0.320)	-0.276 (0.203)
**SCQ**	-0.171 (0.459)	-0.361 (0.107)	-0.025 (0.917)	-0.004 (0.987)	0.058 (0.820)

*ADOS* Autism Diagnostic Observation Schedule, *MSEL* Mullen Scale of Early Learning, *VABS* Vineland Adaptive Behaviour Scales, *SCQ* Social Communication Questionnaire, *SDS* Standard Difference Score, *RSDS* Restrained Standard Difference Score, *RDDS* Restrained Duration Difference Score, *RT* Response Time

Text in bold indicates *p*-values < 0.05. ^*^Indicates *p*-values that would be significant at the 0.002 level

## Discussion

The primary aim of this study was to examine the utility of an eye-tracking paradigm as a physiological index of RJA behaviours in children with and without a current diagnosis of ASD. Although previous eye-tracking studies during live interaction have shown that eye movement, head movement or both may affect the RJA behaviours of high-risk children [22], most existing eye-tracking studies that used pre-recorded stimuli have not examined this effect. Furthermore, the current study, to the best of our knowledge, is the first to examine this specific effect in a cohort of preschool children aged 3 to 6 years. Previous studies have shown group differences in RJA behaviours in infants [22, 31, 39] and toddlers [25]. Contrary to previous studies with a similar age range [24, 41], our results showed significant differences in RJA behaviours between ASD and TD preschool-aged children in an eye-tracking paradigm using pre-recorded stimuli. Our results support another study that found reduced gaze following accuracy in ASD children [18]. This follows from the literature suggesting that difficulties in RJA behaviours emerge early in life and become progressively evident later in life.

In the present study, gaze accuracy in TD children was more accurate on trials where more information was available (specifically, gaze accuracy was increased on Eyes/Head trials versus Eyes-Only trials). There was only a trend for this same pattern in children with ASD (i.e., a trend for increased accuracy for Eyes/Head trials versus Eyes-Only trials), and the pattern was statistically stronger in TD children. These results partially support other published work where participants with a high risk of ASD or participants with ASD failed to use the information encoded in the eye movements of other people during RJA tasks in a live interaction [22] and pre-recorded stimuli [43]. This finding is also in line with studies suggesting that children on the spectrum pay less attention to eyes [28, 57–65] and have difficulties in interpreting eye information [66, 67]. In the current study, neurotypical children’s gaze accuracy was higher when more joint attention information was available to them (i.e., gaze accuracy was improved for the Eyes/Head condition in comparison to the Eyes-Only condition). This suggests that the children without a diagnosis of ASD were better able to utilise various sources of social and interpersonal communicative information.

A reduced ability to engage in joint attention is expected to influence a child’s later development in several domains. For example, during language development, children must be able to associate an object and the relevant word for the object [6]. Therefore, reduced gaze accuracy can negatively influence the ability to learn new words. Joint attention is also important in non-verbal communication and socio-cognitive development. In our study, correlational analyses revealed reliable associations between various eye-tracking measures and clinical information. In line with previous research [18], SDS was positively associated with parent-reported Communication and Socialisation scores in children with ASD. In addition, gaze following accuracy was positively correlated with VABS communication scores. In other words, more accurate gaze profiles were associated with higher social and communication scores. Our study also linked eye-tracking measures to standardised measures of cognition. In particular, MSEL scores were positively correlated with numerous eye-tracking measures in children with ASD. This suggests that children with ASD who have better early learning and more adaptive behaviours (as per parent reports) are more likely to follow or respond to bids of joint attention. In addition, SCQ scores were negatively correlated with different eye-tracking measures in children with ASD. ADOS calibrated severity scores and scores on the social affect scale were also negatively correlated with various eye-tracking measures. Clinically, these findings suggest that more severe symptomatology were associated with less accurate gaze responses to requests for joint attention. Collectively, the correlations provide support for the notion that eye-tracking variables may provide utility as biomarkers for ASD [68]. Of course, it is important to highlight that these cross-sectional, correlational analyses cannot speak to causation.

The finding that clinical measures were consistently correlated with eye-tracking variables, in the direction expected, suggests that the ASD children’s eye-tracking responses are reflective of their functioning and associated difficulties. In this regard, if a pre-schooler fails to follow the non-verbal cues of communication, that behaviour may adversely impact social learning. Joint attention could therefore serve as a target for early intervention programs.

In the current study, there were no significant correlations between eye-tracking data and clinical characteristics for TD children, presumably due to the restricted range of clinical scores for TD children, so that eye tracking is not a useful biological or diagnostic marker (as there is no deficit to detect). The ASD group of children is likely more heterogenous than the TD group. This heterogeneity is a further reason why it is helpful to have a reliable biomarker by which to track specific difficulties, such as in RJA. It is clinically important to keep in mind that all children with ASD will not process information in the same way, and therefore will respond to interventions differently.

The current study differs from a study reported previously [16]. Franchini, et al. [16] included a younger cohort of children with ASD (mean age 2.8 years) and found no significant correlations between gaze following accuracy and clinical measures. However, they reported differences in RJA based on task conditions. In that study, RJA was improved when the stimulus was intense, and when supported by gestural pointing [16]. The findings of Thorup, et al. [22] are similar to our findings. They also found a significant reduction in gaze accuracy of high-risk infants in an Eye-Only condition (during a live eye-tracking interaction). Our results suggest that pre-schoolers with ASD are more likely to respond to joint attention when more visual information is available (although this result must be tempered as it was only trending towards significance). Exaggerating or augmenting content cues might help preschool children with ASD in RJA tasks in the context of early intervention. Given that the mean age of the Franchini, et al. [16] cohort was 2.8 years and our cohort was 4.6 years, it appears that this preschool-age period might provide a good time when gaze following accuracy and other eye-tracking measures could be used as an adjunct in the ASD diagnosis process, with a particular focus on improving our understanding of the underlying mechanisms involved in how early intervention may improve RJA in ASD. Furthermore, the numerous significant associations between physiological eye-tracking measures and clinical severity could potentially help individualise treatment for children with ASD: it is not hard to imagine that future interventions could be individualised based on not only the clinical and behavioural characteristics but also the physiological indices of information processing such as the child’s unique eye-tracking profile.

### Limitations

Despite the utility of the current study, there are several limitations to keep in mind. First, there was a gender skew towards males in the ASD group, as would be clinically expected. Nevertheless, further studies with more female participants are required to clarify our results, as differences in autism presentation and diagnosis between males and females have been documented [69]. For example, studies have shown that girls on the spectrum behave similarly to neurotypical boys and girls on certain socially orientated tasks: for example, girls demonstrate enhanced attention to faces during scenes that do not have social interactions [70, 71]. In addition, TD men with high autistic-like traits exhibit worse accuracy of gaze shifts, while TD women have similar eye-gaze following behaviour regardless of autistic-like traits [72]. A follow-up study exploring the contribution of biological sex to joint attention behaviours in ASD is therefore indicated.

Further, the participant groups also differed in sample size, with the ASD group being three times as large as the TD group. The ASD participants in this study were recruited from an ASD-specific centre and there was good uptake to the study. Despite significant efforts of the team to recruit control participants, there was less interest from the families of neurotypical children at the centre to participate in the study, which is probably not surprising given the study is less meaningful for children without a developmental diagnosis.

It is also useful to note that the participant groups were matched on chronological age but not on developmental abilities. This may have accentuated the main results of this study, particularly the observed significant group differences and correlations between different eye-tracking measures and different clinical information in the ASD group. Further studies with larger sample sizes with a developmentally age-matched group are suggested to confirm this finding.

As reported in the Methods, children with ASD were not excluded from the study if they had a comorbid diagnosis. Although this has implications for any strict interpretation of the findings reported here, the inclusion of co-morbid conditions in ASD research is ecologically valid. Indeed, it is rare in clinical practice to encounter a young person who has a ‘pure' autism spectrum diagnosis with no other psychiatric or developmental comorbidities.

Moreover, it is important to consider the limitations due to the pre-recorded nature of the stimuli. In this work, we aimed to determine whether such stimuli can help identify differences in RJA behaviours in ASD and TD preschool children and determine possible correlations between the derived eye-tracking measures and clinical information. The results in this study suggest that differences in certain eye-tracking measures exist in the context of the stimuli used in this study. However, we acknowledge that it is not as ecologically valid as a live interaction task where an actor may exaggerate/augment their cues and even have multiple attempts to initiate joint attention. In comparison, the actor made no exaggerated cues in both the Eyes-Only and Head/Eyes conditions, as illustrated in Fig. 1. Future research should compare the presence and absence of exaggerated and pre-recorded movements in these two conditions for a more ecologically valid scenario.

Finally, given the cross-sectional nature of the study, it is not possible to infer any causative mechanisms. For example, it is not clear whether adaptive functioning may lead to improved social engagement, as reflected by gaze accuracy, or whether the development of gaze accuracy may help improve adaptive behaviours. In addition, it is not clear whether the observed eye-tracking profile is the result of differences in abilities or due to the lack of interest and motivation in engaging in social interactions and following gaze. However, the association between these measures is clinically important. From a clinical perspective, the finding suggests that eye-tracking technology could be used as a biomarker of adaptive functioning in young children, and could potentially be implemented into a diagnostic test battery, or as a measure of treatment progress. This will have implications for targeting the intervention, in terms of skills building versus increasing interest and engagement in social-communicative tasks. Future studies are indicated for future exploration of this issue.

## Conclusion

In this study, we found that there are differences in the RJA behaviours between ASD and TD preschool children. In addition, we found that several eye-tracking measures of RJA behaviours in preschool children with ASD are associated with different clinical measures commonly used to diagnose ASD.

## Data Availability

The datasets generated and/or analysed during the current study are not publicly available due to privacy concerns but are available from the corresponding author on reasonable request.

## Abbreviations

ADOS: Autism diagnostic observation schedule
AE: Age equivalent
AOI: Area of interest
ASD: Autism spectrum disorder
DSM: Diagnostic and statistical manual of mental disorders
DQ: Developmental quotient
ESCS: Early social communication scale
HR: High risk
IQ: Intelligence quotient
LR: Low risk
MSEL: Mullen scale of early learning
RJA: Response to joint attention
RDDS: Restrained duration difference score
RSDS: Restrained standard difference score
RT: Response time
SCQ: Social communication questionnaire
SDS: Standard difference score
SRS: Social responsiveness scale
TD: Typical development
VABS: Vineland adaptive behaviour scales
